# Validity and Reliability of the Greek Version of the Psychosocial Impact of Dental Aesthetics Questionnaire (Gr-PIDAQ)

**DOI:** 10.3390/dj14010014

**Published:** 2026-01-01

**Authors:** Chrysanthi Anagnostou, Ioannis P. Zogakis, Ilias Pagkozidis, Apostolos Matiakis, Ilias Tirodimos, Theodoros Dardavesis, Zoi Tsimtsiou

**Affiliations:** 1 Department of Hygiene, Social-Preventive Medicine and Medical Statistics, School of Medicine, Aristotle University of Thessaloniki, 54124 Thessaloniki, Greece; 2 Department of Orthodontics, School of Dentistry, Aristotle University of Thessaloniki, 54124 Thessaloniki, Greece; 3 Department of Oral Medicine and Pathology, School of Dentistry, Aristotle University of Thessaloniki, 54124 Thessaloniki, Greece

**Keywords:** psychometric properties, quality of life, dental aesthetics, malocclusion, PIDAQ

## Abstract

**Background/Objectives**: The Psychosocial Impact of Dental Aesthetics Questionnaire (PIDAQ) is a useful tool for investigating the psychosocial impact of malocclusion and dental aesthetics on the quality of life of young adults. This study aimed at developing a culturally adapted Greek version, assessing its validity and reliability. **Methods**: The questionnaire underwent bilingual translation, followed by cultural adaptation with 10 debriefing interviews. Gr-PIDAQ along with the aesthetic component of the Index of Orthodontic Treatment Need (IOTN-AC) were completed by 270 young adults for the evaluation of its psychometric properties. Fifty dentists also participated, assessing its face validity. **Results**: Confirmatory factor analysis verified the four-factor structure of the original version of PIDAQ. The ability of Gr-PIDAQ to discriminate between individuals according to the perceived need for improvement in dental appearance was confirmed (*p* < 0.001). Participants exhibiting greater malocclusion severity as assessed by the IOTN-AC presented higher PIDAQ scores (*p* < 0.001). Face validity was confirmed by 99.6% of participants and 96% of dentists. Cronbach’s alpha coefficient of the overall scale was 0.94 (the four subscales ranged from 0.78 to 0.9), while excellent repeat measurement agreement was detected (ICC = 0.95, *p* ˂ 0.001). **Conclusions**: Our findings suggest that Gr-PIDAQ is a valid and reliable tool that can be used to measure orthodontics-related quality of life in Greek speaking adults. In terms of clinical application, it can be used to assess the orthodontic treatment need and record patients’ perspective both before orthodontic treatment initiation and later during the assessment of its effectiveness, serving as a Patient-Related Outcome Measure (PROM).

## 1. Introduction

Based on the definition of oral health-related quality of life (OHRQoL), oral health is increasingly recognized as encompassing more than merely the absence of disease and functional impairment. It also includes the effects of oral health conditions on individuals’ psychosocial well-being, social life and dentofacially-related self-confidence [1]. Thus, OHRQoL refers to the impact of oral health or disease on one’s daily functioning, well-being and overall quality of life [1].

Orthodontic abnormalities are oral health-related conditions that may influence an individual’s quality of life. Not only may they greatly affect oral function, but they may impact social acceptance and self-esteem as well, due to their functional and aesthetic implications, as they compromise both occlusion and smile architecture [2,3]. The main goal of orthodontic treatment has traditionally been to correct malocclusion and improve dental aesthetics. Often, the motivations for seeking orthodontic treatment are psychosocial and extend beyond the diagnostic measurements of clinical parameters performed by orthodontic practitioners [2,4]. Therefore, for a comprehensive orthodontic evaluation, the use of tools that measure psychosocially-related variables is essential. It is of primary importance that clinicians do not focus exclusively on existing occlusal anomalies, but also consider factors related to each patient’s mindset [5,6].

Due to the importance of the OHRQoL concept, a multitude of tools measuring QoL have been developed. The Psychosocial Impact of Dental Aesthetics Questionnaire (PIDAQ), a self-rating questionnaire investigating the effect of malocclusion and dental aesthetics in the quality of life of young adults, developed in 2006, is one of the most extensively applied tools in clinical and research settings [7]. To the authors’ best knowledge, although several translated and validated PIDAQ versions have since been published [8,9,10,11,12,13,14,15,16], no culturally adapted Greek version is yet available.

Therefore, the aim of this study was to develop a valid and reliable Greek version of PIDAQ, allowing the investigation of the psychosocial impact of malocclusion and dental aesthetics on the quality of life of young adults. This characteristic distinguishes the index from other measurement tools and scales commonly employed by Greek orthodontists, which predominantly rely on objective clinical assessments, such as occlusal or skeletal parameters, or are designed to evaluate populations within different age groups. In this respect, the index offers a distinct evaluative perspective by addressing dimensions that extend beyond purely clinical findings and by targeting a population segment not adequately represented in the existing instruments.

## 2. Materials and Methods

### 2.1. Development of the Gr-PIDAQ

Prior to distributing a questionnaire to a population with dissimilar cultural context, translation and cultural adaptation procedures are necessary, as proposed by the International Quality of Life Assessment Project [17]. For the purposes of the study, permission to translate and use PIDAQ was obtained by the manufacturers. The guidelines of the International Organization for Pharmacoeconomics and Outcomes Research were followed for the translation process [18].

### 2.2. Bilingual Translation of PIDAQ

Forward Translation. PIDAQ was independently translated into Greek by one dentist and one orthodontist, proficient in English and Greek. Following consensus, the initial draft of the translated version was formed.

Back translation. The Greek translation was translated back into English by a professional translator uninformed of the original scale. After back translation, comparison with the original questionnaire and reconciliation of differences, the Greek version was formed [19].

### 2.3. Cultural Adaptation

Debriefing interviews were carried out in a convenience sample of 10 volunteers (five female and five male students). Participants completed the tool, while commenting on their understanding of the scale and each individual statement, highlighting both elements possibly causing confusion and difficulty in comprehension, as well as concepts requiring different ways of rendering in Greek. Apart from translating the original PIDAQ, semantic and conceptual equivalence were assessed in comparison to the original questionnaire leading to the formation of Gr-PIDAQ. All items retained their original content and no additional cultural modifications were necessary beyond the translation and equivalence assessment.

### 2.4. Study of Psychometric Properties

Participants were young adults with an age range from 18 to 30 years old, approached at the Central Library of the Aristotle University of Thessaloniki Campus from August to November 2023. The desired sample size should consist of at least 230 young adults; ten participants for each of the 23 scale items [20]. Participants were orally informed about the study purposes by a dentist who was a member of the research team, and asked to participate voluntarily and anonymously, upon reading the information leaflet and signing the informed consent form. Prior to the administration of the questionnaire, students to be included were asked about history of previous orthodontic treatment. Individuals with moderately to severely discolored anterior teeth, missing or fractured teeth, cavities or extensive restorations on the anterior teeth, craniofacial anomalies, and previous or on-going orthodontic treatment were excluded. Dentists and dental students were also excluded due to knowledge and expertise in the study field. No incentives were given to increase participation rate. Additionally, a total of 50 dentists were asked to participate in the study as experts with knowledge of the field. Participation was voluntary and a signed informed consent form was required. They were asked to comment on whether the scale is valid in measuring its concept, to test the face validity of Gr-PIDAQ.

The study tool included PIDAQ, a 23-item psychometric scale, requiring individuals to rate the impact of dental aesthetics on a five-point Likert scale ranging from 0 to 4 (“not at all”, “a little”, “somewhat”, “strongly” and “very strongly”, respectively). PIDAQ consists of four subscales: Dental Self-Confidence (six statements), Social Impact (eight statements), Psychological Impact (six statements) and Aesthetic Concern (three statements). Items in some domains are reversed-scored to produce a consistent measure of impact and to facilitate interpretation of results. Higher PIDAQ scores are indicative of lower satisfaction with the present dental condition [7]. IOTN-AC was also used to assess participants’ perception of dental esthetics and severity of malocclusion. It consists of ten photographs of anterior teeth which display various degrees of malocclusion. After studying these photographs and without time limitation, each participant was asked to indicate the one most closely resembling their own dentition [21]. The study tool also comprised demographic questions and was pilot tested in a convenience sample of 15 participants who met the study inclusion criteria. During pilot-testing, recording feedback on the participants’ understanding of the tool’s questions did not reveal any comprehension issues, confirming propriety for use in the study that followed.

### 2.5. Ethics Approval

To conduct the study, permission was obtained from the Bioethics Committee of the Department of Medicine of the Aristotle University of Thessaloniki (Protocol No. 208/2023/14 July 2023). An informed consent form was obtained from all participants.

### 2.6. Statistical Analysis

Statistical analyses were conducted using the Jamovi software (Version 2.3). The Shapiro–Wilk test was used to examine the continuous variables’ distribution. Data was not normally distributed; therefore, non-parametric tests were applied. The reliability of the scale was determined by internal consistency testing using Cronbach’s alpha coefficient. Test-retest reliability was assessed using intraclass correlation coefficient (ICC). Regarding construct validity, confirmatory factor analysis was applied with a maximum likelihood procedure. Suitability of data was tested with the Kaiser–Mayer–Olkin (KMO) test and Bartlett’s test of sphericity. To assess the fit of the proposed model, the following indices were used: the quotient of χ^2^ with the degrees of freedom (χ^2^/df), the Comparative Fit Index (CFI), the Tucker–Lewis index (TLI), the Root Mean Square Error of Approximation (RMSEA) and the Standardized Root Mean Square Residual (SRMR). Known groups validity was tested using Mann–Whitney tests to compare PIDAQ scores with participants’ gender and perceived need for improvement of dental image. Criterion validity was tested using Kruskal–Wallis test comparing the total PIDAQ and subscale scores with the IOTN-AC score. Confirmation of face validity was based on descriptive analysis of participants’ and dentists’ response to the study’s instrument. Finally, the chi-squared test was used to compare qualitative, independent variables. Significance level was set at 0.05, two-sided (*p* < 0.05).

## 3. Results

A total of 270 students participated in the study (79.9% response rate). Thirty-nine of those approached declined participation, whereas 29 declined completion of the informed consent form. Participants had a median age of 21 years (IQR:20, 23) and 55.2% were female (n = 149). Among them, 57% (n = 154) believed that the aesthetic appearance of their dentition required enhancement.

### 3.1. Validity Tests

#### 3.1.1. Confirmatory Factor Analysis

Data suitability for confirmatory factor analysis was tested. Specifically, the Kaiser-Meyer-Olkin (KMO) test indicated excellent sampling adequacy (KMO = 0.93), while Bartlett’s test of sphericity test confirmed that correlations between variables were sufficient for factor analysis (χ^2^ = 3523, *p* < 0.001). Confirmatory factor analysis with a maximum likelihood procedure was applied to confirm the model proposed by PIDAQ developers. Table 1 and Table 2 demonstrate the factor loadings of the variables and the factor covariances. Data satisfactorily confirm the four-factor model proposed by the manufacturers, as emerged from the model fit study. More specifically: χ^2^/df = 2.625 (*p* < 0.001), indicating a good fit, CFI = 0.89 and TLI = 0.88, both suggesting marginal fit, RMSEA = 0.078 (90% CI:0.07, 0.085) and SRMR = 0.065, both indicating a good fit. As three indicators display a good fit to the data, with the rest indicating a marginal fit, we concluded that Gr-PIDAQ confirms the factor structure proposed by its developers.

#### 3.1.2. Known Groups Validity

##### Improvement of Teeth Image

Participants were divided into two groups based on whether they believed the aesthetic appearance of their dentition needed enhancement. Participants in the perceived need group had higher total PIDAQ and subscales scores when compared to those perceiving no need (Table 3). This suggests that those who felt their teeth needed improvement experienced a greater impact on their oral health-related quality of life, as reflected by higher overall PIDAQ scores and higher scores on all subscales compared to those who felt no improvement was needed.

##### Gender

Νo significant difference in scores between male and female participants was detected (median 18 versus 19, respectively, *p* = 0.51).

#### 3.1.3. Criterion Validity

The median scores of total PIDAQ and its four subscales were statistically significantly higher in the groups with increasing severity in terms of presence of malocclusion according to IOTN-AC scores, as presented in Table 4. This means that individuals with more pronounced malalignment, as indicated by IOTN-AC, experienced a greater impact on their oral health-related quality of life, as marked by PIDAQ scores, and this difference was statistically significant.

#### 3.1.4. Face Validity

A 10-point Likert scale was used to obtain participants’ assessments of Gr-PIDAQ’s success in capturing the intended concept. Two hundred and sixty-nine participants (99.6%) agreed and evaluated the degree of achievement with a median of 8 (IQR:7, 9). Additionally, 50 dentists were approached, with a median age of 32 years (IQR:28, 39.8) and a median work experience of 6 years (IQR:4, 12.8). The majority of them (n = 48, 96%) agreed with the above statement, with their evaluations yielding a median score of 10 (IQR:9, 10), indicating high perceived achievement.

### 3.2. Reliability Tests

#### 3.2.1. Internal Consistency Reliability

Gr-PIDAQ demonstrated high internal consistency (Cronbach’s coefficient alpha = 0.94). Additionally, each subscale’s internal consistency was satisfactory: α_DC_ = 0.9, α_SI_ = 0.78, α_PI_ = 0.85 and α_AC_ = 0.87.

#### 3.2.2. Test-Retest Reliability

Participants in the first phase of the study were asked to complete the same scale again after a two- to four-week interval. In total, 120 individuals provided their contact information for that purpose and 70 completed it again voluntarily and anonymously. Results of the agreement indicate high test-retest reliability: ICC = 0.95 (95%CI:0.92, 0.97, *p* ˂ 0.001).

## 4. Discussion

This study aimed at providing a valid and reliable version of PIDAQ in the Greek language, enabling the investigation of the psychosocial effects of dental appearance and malocclusion on the quality of life of Greek young adults. The translation and cultural adaptation process yielded findings that confirm the reliability and validity of Gr-PIDAQ. Evidence of validity was established through tests of construct validity (confirmatory factor analysis, known-groups method, criterion validity) as well as through an assessment of face validity. Specifically, confirmatory factor analysis verified the four-subscale factor structure originally proposed by the developers of the instrument, demonstrating that it is retained in the Greek version. Using the known-groups method, it was demonstrated that the Greek version successfully produces statistically significantly higher scores among individuals with desire for improvement of dental appearance and those with high scores on the IOTN-AC index. Face validity was confirmed by the overwhelming majority of both study participants and a panel of expert dentists. The reliability of the Greek version was assessed through internal consistency of the overall scale and its subscales, as well as through test-retest reliability, all of which were found to be high.

The psychometric properties of Gr-PIDAQ were comparable to those of the original scale [7] and its available validated versions, as reported in the literature [8,9,10,11,12,13,14,15,16]. This indicates that the Greek version effectively measures the impact of dental appearance on quality of life in a way that is consistent with the original questionnaire and other adaptations. In particular, all previous versions similarly confirmed the scale’s construct validity, and reported good/high internal consistency and test-retest reliability [8,9,10,11,12,13,14,15,16], attesting to the robustness of the instrument across diverse populations. Moreover, in the Chinese, Nepalese and Croatian adaptations [9,10,11], no significant effect of gender on PIDAQ scores was observed, suggesting that self-perceptions of dental aesthetics are generally similar for males and females. Additionally, these and other adaptations, including the Italian [12], Arabic [13] and Indian [14] versions, demonstrated the instrument’s capability to distinguish between different levels of malocclusion, reflecting its sensitivity to variations in orthodontic treatment need across diverse populations. In addition, the Nepalese version [10] incorporated face validity assessments with an expert panel, reinforcing the importance of cultural adaptation in capturing subjective perceptions of dental aesthetics. Overall, these studies support the robustness of the PIDAQ across diverse populations and confirm that the Gr-PIDAQ performs consistently with prior validations, demonstrating its reliability and applicability in the Greek context.

More specifically, concerning reliability, Cronbach’s alpha coefficient of Gr-PIDAQ was higher than the general requirements of a reliability coefficient for an already established scale (α > 0.8) [22], higher than the original study’s coefficient [7] and comparable to those of other PIDAQ versions [8,9,10,11,12,13,14,15,16]. Additionally, test-retest reliability was excellent and comparable to those of several translated and validated PIDAQ versions [8,9,10,11,12,13,14,15,16], reinforcing the reliability of Gr-PIDAQ.

In terms of validity, the confirmatory factor analysis conducted for construct validity showed that the four-factor model proposed by the original developers is satisfactorily confirmed for the Gr-PIDAQ as well, indicating that the underlying theoretical structure of the scale is preserved and functions as intended in the Greek population. Furthermore, the instrument exhibited strong known-groups validity, with participants perceiving a need for dental esthetic improvement scoring significantly higher on both total and subscale PIDAQ measures than those who did not, corroborating prior research [12], confirming that the questionnaire can effectively capture differences in perceptions of dental aesthetics, discriminating between groups with different perceptions of dental aesthetics, reflecting its ability to measure meaningful differences in self-perceived oral appearance. Consistent with findings from the original PIDAQ development [7] and various validated versions, including the Chinese, Nepalese and Arabic adaptations [9,10,13], gender was not found to exert a significant effect on PIDAQ score. In line with earlier research, gender was not identified as a significant determinant of satisfaction with dental appearance [23,24,25,26], suggesting that perceptions of oral aesthetics are largely independent of it. This indicates that the Gr-PIDAQ functions equivalently for both males and females, supporting its broad applicability and reinforcing its reliability as a measure of self-perceived dental aesthetics across genders. Nonetheless, evidence suggests that adult women tend to exhibit greater concern and dissatisfaction pertaining to their dental aesthetics, which may account for their comparatively higher demand for orthodontic care [27,28,29,30].

In this study, IOTN-AC, commonly used worldwide, was implemented as standard to verify the criterion validity of Gr-PIDAQ. Results indicated that the Greek version could differentiate IOTN-AC scores, an attribute underscored in the original PIDAQ version [7] and several other studies [9,10,11,12,13,14,16,26,31]. Specifically, the Greek version was able to distinguish between varying levels of malocclusion, with participants with more pronounced malalignment reporting higher scores on the questionnaire, underscoring the instrument’s sensitivity to differences in orthodontic need and further supporting its validity and effectiveness in capturing the psychosocial impact of dental aesthetics in the Greek population.

Regarding the scale’s face validity, two assessments were conducted to capture the perspectives of both participants and dental professionals. Young adults and field experts alike confirmed Gr-PIDAQ’s ability to measure its concept of dental aesthetics-related quality of life. Face validity reflects the extent to which an instrument appears to measure what it is intended to measure, and the excellent face validity observed in our study indicates that participants found the items clear, relevant and representative of the construct being assessed [32]. In comparison, face validity testing was performed solely on the Nepalese validation study by an expert panel [10], highlighting that our approach also incorporated the views of the target population, strengthening the overall evaluation of the scale.

Overall, the Gr-PIDAQ demonstrated excellent psychometric properties. Unlike many indices and scales commonly used by Greek orthodontists—which primarily focus on objective clinical findings, such as the severity of malocclusion, treatment need, or case complexity—or those designed for different age groups, Gr-PIDAQ specifically captures the subjective perceptions and emotional responses of young adults regarding their dental aesthetics. By addressing these psychosocial dimensions, the Gr-PIDAQ functions as a robust patient-reported outcome measure (PROM), providing insights that cannot be obtained through clinical examination alone. As such, it offers a complementary tool that enriches orthodontic assessment by illuminating how malocclusion, apart from leading to complications related to normal functional occlusion, also affects dental aesthetics, everyday life, social interactions and self-esteem within this population. Incorporating PROMs like the Gr-PIDAQ aligns with contemporary principles of patient-centered care, which emphasize understanding and valuing the patient’s own perspective on their health, well-being and treatment priorities [6]. The use of the Gr-PIDAQ therefore supports more holistic treatment planning, fostering communication and shared decision-making and ultimately improving the alignment of orthodontic care with the experiences and expectations of young adult patients.

### Strengths and Limitations

The Greek version of the Psychosocial Impact of Dental Aesthetics Questionnaire (PIDAQ) provides a valuable tool for advancing oral health surveillance and epidemiologic research in Greece and other Greek-speaking populations. By offering a culturally and linguistically validated instrument, Gr-PIDAQ enables the systematic assessment of the psychosocial effects of dental aesthetics on an individual’s quality of life, an increasingly important dimension of oral health outcomes. Its integration into epidemiological surveys can help identify population groups most affected by aesthetic-related oral health issues, monitor changes in oral health perceptions over time and inform public health strategies aimed at improving access to aesthetic and preventive dental care. Furthermore, the availability of a validated Greek version facilitates cross-cultural comparisons, contributing to a more comprehensive understanding of how psychosocial factors influence oral health globally. A key strength of the study is its community-based population, as it reflects real-world conditions, increases external validity and supports the generalizability of our findings beyond clinical settings.

However, this study also has certain limitations, one of which arises from the nature of the tool itself. As PIDAQ is not based on participants’ clinical examination, but is rather a self-rating scale, risks of truth concealing exist. Although this is a known limitation of studies using self-rating scales, it does not limit their value and importance [33]. Another limitation is the relatively small representation of individuals with severe malocclusion, with IOTN-AC ≥ 4 (10.4%), since in countries with easy access to orthodontic treatment, it is difficult to gather a large enough sample of individuals with a high degree of malocclusion in this age group. However, the percentage achieved in our study was considered satisfactory, considering it allowed the detection of statistically significant differences among the subgroups and was similar to those found in the study of the original PIDAQ (8.8%) [7] and other studies [34]. In addition, although the Greek version of PIDAQ was developed through a rigorous bilingual translation and cultural adaptation process, formal content validity assessment using a larger panel of experts and calculation of the Content Validity Index (CVI) was not performed; this should be addressed in future studies to further strengthen the tool’s validity.

## 5. Future Directions

The valid and reliable newly developed version of the PIDAQ carries several important implications for future research and clinical practice. It provides a valuable foundation for further investigations into the psychosocial dimensions of orthodontics-related quality of life. Clinically, its application extends beyond the initial assessment of orthodontic treatment needs, enabling practitioners to capture and monitor patients’ subjective experiences throughout the treatment trajectory. This positions the instrument as a useful Patient-Reported Outcome Measure (PROM), facilitating the systematic evaluation of therapeutic outcomes and the overall quality of care in orthodontic practice. Moreover, the integration of such patient-centered measures into clinical and research settings may enhance the evidence base guiding orthodontic interventions. Future studies should aim to further validate the instrument’s applicability across diverse populations and treatment contexts.

## 6. Conclusions

The newly developed Greek version of PIDAQ demonstrated excellent psychometric properties. Consequently, it is a promising tool that can be used for further research on orthodontics-related quality of life. From a clinical standpoint, the instrument may be employed not only to determine the need for orthodontic intervention but also to systematically document patients’ subjective perspectives both at baseline, prior to the initiation of treatment and subsequently during the evaluation of therapeutic outcomes, thereby serving as a potential Patient-Reported Outcome Measure (PROM) in the context of evaluating the quality of the provided dental services. Further investigation in this direction is needed. Finally, Gr-PIDAQ could serve as a means of documenting the benefits of orthodontic treatment in the context of epidemiological studies in Greek-speaking adult populations, thereby informing the development of data-driven health policies and the optimization of dental service provision.

## Figures and Tables

**Table 1:** Factor loadings per item of Gr-PIDAQ.

Factor	Indicator	Estimate	SE	Z	Standardized Estimate	*p*-Value
DC	P1 *	0.71	0.04	16.18	0.82	<0.001
	P2 *	0.76	0.05	14.14	0.75	<0.001
	P3 *	0.76	0.04	17.39	0.86	<0.001
	P4 *	0.66	0.05	13.75	0.74	<0.001
	P5 *	0.75	0.05	16.14	0.82	<0.001
	P6 *	0.63	0.05	11.87	0.66	<0.001
SI	P7	0.64	0.06	10.00	0.60	<0.001
	P8	0.51	0.05	10.60	0.63	<0.001
	P9	0.47	0.04	10.76	0.63	<0.001
	P10	0.36	0.03	11.11	0.65	<0.001
	P11	0.40	0.05	7.82	0.48	<0.001
	P12	0.30	0.05	5.81	0.37	<0.001
	P13	0.59	0.06	9.86	0.59	<0.001
	P14	0.55	0.06	10.05	0.60	<0.001
PI	P15	0.83	0.07	12.38	0.69	<0.001
	P16	0.36	0.05	7.96	0.48	<0.001
	P17	0.75	0.05	14.80	0.78	<0.001
	P18	0.69	0.06	12.02	0.67	<0.001
	P19	0.66	0.04	15.27	0.80	<0.001
	P20	0.84	0.06	13.99	0.75	<0.001
AC	P21	0.59	0.05	12.26	0.68	<0.001
	P22	0.96	0.05	19.67	0.93	<0.001
	P23	0.90	0.05	19.69	0.93	<0.001

* Questions with negative content, reverse coded before analysis. DC: Dental Self-Confidence; SI: Social Impact; PI: Psychological Impact; AC: Aesthetic Concern.

**Table 2:** Factor covariances of Gr-PIDAQ.

		Estimate	SE	Z	*p*-Value
DC	DC	1.00 *			
	SI	0.65	0.05	13.7	<0.001
	PI	0.77	0.03	23.3	<0.001
	AC	0.67	0.04	17.0	<0.001
SI	SI	1.00 *			
	PI	0.83	0.03	24.5	<0.001
	AC	0.76	0.04	20.2	<0.001
PI	PI	1.00 *			
	AC	0.79	0.03	25.5	<0.001
AC	AC	1.00 *			

* Fixed parameter. DC: Dental Self-Confidence; SI: Social Impact; PI: Psychological Impact; AC: Aesthetic Concern.

**Table 3:** Median and interquartile range of Gr-PIDAQ and subscales scores of the perceived “need for improvement in dental appearance” group versus the perceived “no need” group.

PIDAQ Scores	Perceived “Need” (n = 154)	Perceived “No Need” (n = 116)	*p*-Value *
DC	10.5 (8, 13.8)	7 (5, 10)	<0.001
SI	4 (2, 8)	2 (0, 4)	<0.001
PI	8 (5, 11)	3 (1, 5)	<0.001
AC	2 (0, 5)	0 (0, 1)	<0.001
Total PIDAQ	25 (17, 36)	13 (8, 18)	<0.001

* Mann-Whitney U test. DC: Dental Self-Confidence; SI: Social Impact; PI: Psychological Impact; AC: Aesthetic Concern.

**Table 4:** Median and interquartile range of Gr-PIDAQ and subscales scores in relation to the four IOTN-AC groups.

IOTN-AC Group	PIDAQ Scores				
	DC	SI	PI	AC	Total PIDAQ
1 (n = 126)	7 (5, 10)	2 (0, 3.8)	3 (1.3, 6)	0 (0, 1.8)	13 (9, 19)
2 (n = 52)	10 (7, 12)	2 (1, 5)	5 (3, 9)	1 (0, 3)	18 (12.8, 30.3)
3 (n = 64)	10.5 (8, 13)	4 (2, 8)	7 (5, 11)	3 (0, 4.3)	24 (18, 36.8)
≥4 (n = 28)	14 (11, 17.3)	9 (5, 10.5)	11 (9, 14)	5 (2.8, 7.3)	36.5 (33, 44.5)
*p*-Value *	<0.001	<0.001	<0.001	<0.001	<0.001

* Kruskal–Wallis test. DC: Dental Self-Confidence; SI: Social Impact; PI: Psychological Impact; AC: Aesthetic Concern.

## Data Availability

The data presented in this study are available on request from the corresponding author due to privacy, ethical and legal restrictions.
